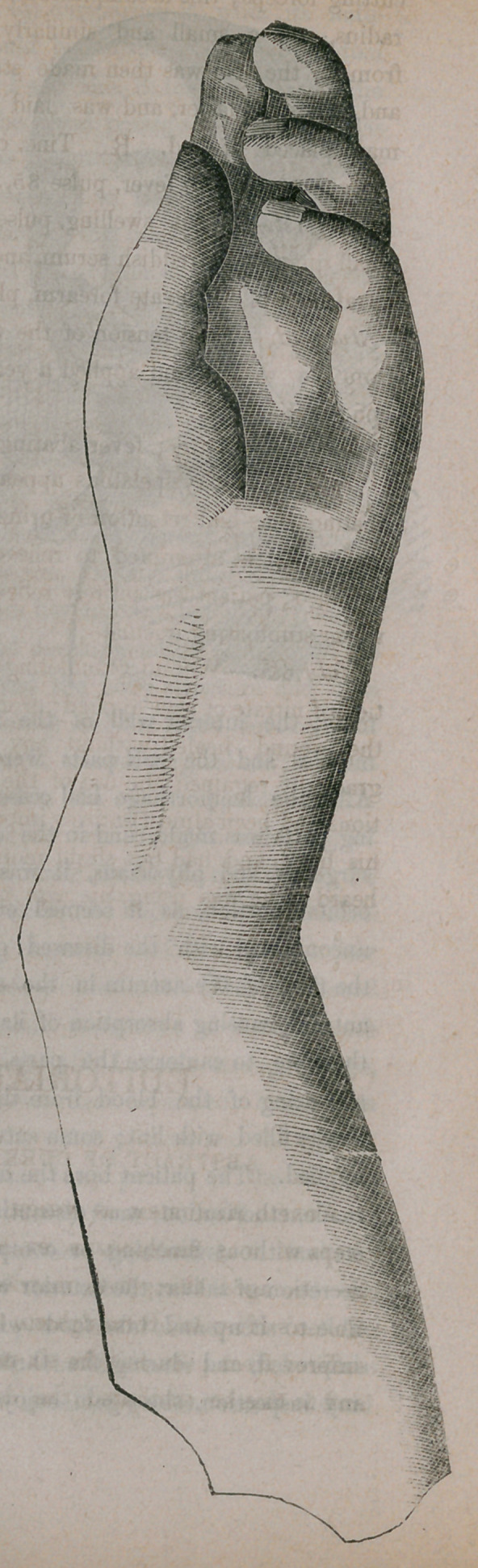# Operative Surgery

**Published:** 1862-01

**Authors:** Charles Winne


### ART. III.—*Operative Surgery.*—By Charles Winne, M. D.

In compliance with my promise to furnish your Journal with a few cases of operations upon bones that have occurred in the course of my practice, I send for your disposal a report of the following cases, which through the artistic skill of a professional friend I had an opportunity of having illustrated, and I think quite truthfully; they are at your service for publication, if you deem them worthy of insertion. I think it can be safely averred that in no department of operative surgery has there been more decided and rapid improvement than in the various operations upon the bones; and certainly when we revert to the limited amount of operative relief that could be furnished in the strumous and cancerous affections of bones; in the deformities resulting from the unfortunate issue of fractures; in caries and necrosis, all of us must feel gratified that in scarcely more than a third of a century, so great advancement has been made in the boldness, skill and dexterous manipulation with which these disorders and conditions are now treated. Much might also be said in view of the great improvement and ingenuity of the mechanical means now employed in the removal of parts deeply imbedded and strongly connected with other related osseous structures, that would have foiled our efforts, if ingenuity had failed in adapting the necessary instruments that are now accessible to all. In this connection we cannot omit the name of Liston, especially as having contributed greatly to the instrumental means whereby the superior maxillary bone is now attacked and removed, wholly or in part. In the early part of this century one of the most distinguished British surgeons, in graphic language, has presented to us the terrible condition of the victim of cancerous disease of the upper jaw, before surgical attempts had been made to relieve the patient. He says, "there is profuse discharge, occasional hemorrhage, and the patient is worn out partly by these causes, partly by misery and anxiety of mind and by starvation, for now he is unable to masticate food, and as the destructive process goes on there is at least great difficulty in swallowing even liquid nourishment, only a small portion of which goes down the throat. I do not know anything more miserable than the death-bed of a patient who dies of this horrible disease." At the period when this was written by Brodie, no case of partial or complete exsection of the upper maxillary bone was known to him except the unsuccessful attempt of Desault. In examining the recorded history of the operations on the superior maxillary bone, I find that mention is made of an attempt by Akoluthus, a physician at Bressau in 1693, to remove a tumor on the upper jaw, that followed the extraction of a tooth. He enlarged the mouth with a cut, removed part of the swelling with four teeth, but not being able to get all around it, he attacked it several times with cutting instruments, and sometimes with the actual cautery, and succeeded in curing his patient.

The names of the distinguished French Surgeons Desault, Dupuytren, Dr. Thomas White, (the latter of whom removed nearly the whole of the superior maxilla,) are honorably mentioned. Gensoul is stated to have first performed the operation of ex-section of the entire superior maxilla in the spring of 1827, without tying the carotid artery, and was not aware at the time, of Lizars' recommendation. Lizars first proposed in 1826 the extirpation of the entire bone, and recommended the plan of first "tying the carotid of the affected side, next making an incision through the cheek, from the angle of the mouth backwards and inwards to the masseter muscle, avoiding the parotid duct, dividing then the lining membrane of the mouth, and separating the soft parts from the bone to the floor of the oribit, detaching the half of the velum palati from the palate bone; having thus divested the bone of its soft coverings, the middle incisive tooth is to be removed, then the one maxillary bone to be separated from the other at the mesial and palatine junctures, and also the one palate bone from the other; then the nasal process should be cut across and its malar process; the eye, with its muscles and cushion being carefully held up by a spatula, the floor of the orbit is to be cleared of its soft connections, and the maxillary bone separated from the lachrymal and ethmord bones with a strong scalpel; the bone is now held by the ptergoid processes, and muscles from which it can be separated with the scissors or forceps." This, which Lizars proposed, he attempted to perform in December, 1827, for a medullary sarcomatous tumor of the antrum, but was obliged to desist in consequence of the excessive hemorrage. This distinguished surgeon again performed two successive operations in 1829 and 1830; in the former he tied first the trunk of the temporal and internal maxillary. In the latter, 1830, he first took up the external carotid. The steps of the operation were similar to those which he first recommended. Partial and entire ex-sections of the superior maxilla were likewise performed with successful results, and the operators became rapidly more bold and skillful.

The name of Deadrich of Rogersville, Tennessee, is first mentioned as entitled to the merit of inaugurating the removal of a portion of the lower jaw of a child of fourteen, who had a tumor occupying the left side of the jaw bone; the wound healed kindly and success was complete.

In the recorded accounts of this operation I find the names of Mott in 1821, and Wardrop in 1827, and successively those of Klein and Delpert, and many noted foreign surgeons and an array of many distinguished names among our own countrymen, as Warren, Stevens, and McClellan, who have shed a brilliant lustre upon this department of operative surgery.

It is due to our respected citizen and colleague, Dr. A. S. Sprague, to mention among the rest a successful operation on the lower jaw which he performed in this city at the Hospital of the Sisters of Charity, I think in 1847. The subject of the operation was a mariner, who had perceived at the root of one of the molar teeth of the right side of the jaw a tumor gradually developing itself for ten years prior to application for relief. When he presented himself for relief the entire half of the jaw bone was involved in the disease; the mass was enormous, both internal and external, extending from the symphysis to the ramus, rendering mastication impossible, and threatening death from inanition, from the increasing difficulty of swallowing even liquids. An incision was commenced at the zygoma and carried along the ramus and base of the jaw beyond the symphysis; another dividing the lower lip and integuments of the chin; the soft parts were rapidly separated, and the facial artery tied, as the bleeding was profuse; by the chain saw the jaw was divided in a healthy portion, and the diseased half was then carefully separated and disarticulated at the joint, using the severed portion to depress the coronord process to detach the temporal muscle, exhibiting clearly the advantage of this mode of procedure. The case terminated favorably, and the new formtion of tissue relieved in a few months the deformity at first caused by the loss of the half of the jaw bone. I fear that already I have encroached too much upon your time and space, and will briefly narrate the history of the two cases that my friend was kind enough to illustrate with diagrams, and of which he took the pains to record himself, or I might not now have had it in my power to recall them accurately to my memory.

A Scotch lady about 40 years of age, had occasion nearly two years before the time she presented herself to me for advice, to have one molar tooth extracted on the left side of the adveolar process, upper jaw bone, for tooth ache, and ulceration about the root of it; the pain continuing and increasing to great severity, and the contiguous tooth apparently being also diseased, was subsequently drawn; the alveola continued to swell gradually, at first so imperceptibly that it was only by comparing it with its anterior period and size, that the patient could be satisfied as to its gradual advancement; about four months before I saw Mrs. B. the swelling of the alveolar process increased rapidly, and the nasal passage of that side was evidently encroached upon, the tumor was becoming distinct from the surrounding parts, was of excessive hardness, and so continued until a few months before I saw her, when the portion in the mouth, covered by the integument became somewhat elastic about the prominent part, bulging out the cheek and feeling somewhat cartilaginous; that which more especially belonged to the alveolar was spongy, vascular, and when bruised by any portion of food, bled readily; the paroted and submucous and cublingual glands exuded an unusual quantity of saliva and mucus; the poor creature suffered severe and almost continued pain, except when partially relieved by anodynes or slight effusion of blood; her alarm was extreme, from the fact of having lost her mother at about the same age as herself, from a cancerous affection of the scalp and skull, in the region, I should judge of the union of the parietal with the occipital bone; from her account her mother suffered more than a year with the growth and development of a tumor on the scalp, that ulcerated and gradually destroyed the bony structure and dura mater. Marked anxiety and physical suffering and the rapid development of the tumor had at length constrained Mrs. B. to seek surgical aid; the external appearance of the disease is portrayed in the cut on opposite page.

During the first steps of the operation she was brought under the influence of an anasthetic; an incision was made from the zygoma to the angle of the mouth, avoiding the parotid duet; the flap was then detached from the upper jaw bone to the level of the orbit and the median line, thus exposing the exterior of the diseased mass; considerable bleeding ensued and rendered the tying of small blood-vessels necessary; an incisor tooth was drawn, and partly by sawing and cutting with Liston's strong forceps, the palatine processes and the palatine bones were separated; it was then discovered while pursuing the section of the nasal process, and the separation from the molar bone, that the exterior bony portion of the antrum was absorbed by the pressure of the tumor, from the inside, and was covered by a dense and exceedingly vascular membrane; it had no connection by bone with the floor of the orbit, which seemed unaffected by the disease; in laying it open close to the floor of the orbit with a sickle shaped strong knife, the character of the disease became apparent, as it rolled out a perfect moulded gelatiniform or colloid mass; the interior wall of the antrum, the attachment to the pterygoid muscles and the soft parts were removed with forceps and scissors, &c. After the hæmorrhage had ceased, a careful exploration of all the remaining parts was made, and in the concurrent opinion of several experienced surgeons and physicians, it was not deemed necessary to remove the orbital portion, as it seemed entirely free from disease; it was in fact unconnected with the diseased growth which had evidently commenced at the floor of the antrum in the alveolar portion, and extended up into the antrum, causing absorption of its external wall; it was thought advisable at the time to cauterize the parts, which was done with the actual cautory; all oozing of the blood from the wound now ceased, and did not return; it was filled with lint; some sutures were introduced, plasters and compass applied. The patient bore the operation well. After the first incisions were made, etherization was discontinued, and she calmly bore the subsequent steps without flinching or complaint. For some days there was profuse secretion of saliva; the exterior wound healed kindly, and in a week she was able to sit up and take food with but little difficulty. Her general health improved, and during the three or four months that she remained under my inspection, she again enjoyed immunity from pain; life ceased to be a torment; the internal excavation finally filled up with granulations, and cicatrized with a healthy membrane, to such an extent that there was little falling in of the cheeks, and the excavation was scarcely apparent. (See cut on page 173.)

I never could obtain a detailed account of the subsequent condition of this lady, as she removed from the city; I learned however, that about a year after her removal from this place, some internal malignant disease terminated her life. The only consolation to be derived from the history of this case, is that for a time, she was relieved by a brief but severe operation from most poignant suffering, and a loathsome and terrible imminent death.

Case Second.—I am indebted to my friend Dr. Ellery P. Smith, formerly of this city, for the drawing of this, as of the former case, and the narrative is in his own words, which I here transcribe:—

*Notes of a Case of Angular Deformity of the Left Forearm.*—Henry Mangers, aged 28, was admitted into the Hospital of the Sisters of Charity, June 24th, 1854, for the removal of a distortion of the forearm, both bones of which had been transversely fractur. ed Feb. 25, 1854. The radius formed the acute angle. On June 29th, Dr. Winnie commenced the operation by an incision upon the most prominent portion of the radius, and having carefully denuded the bone of its investing muscles, he removed a wedge shaped portion of the radius by means of the saw and cutting forceps; this accomplished, the ulna was found in contact with the radius, and a small and similarly shaped piece of bone was removed from it; the arm was then made straight, the wound dressed with sutures and adhesive plaster, and was laid in a well padded wire splint, and the man placed in bed. ℞. Tinc. opii gtt. xl.

*June 30th.*—No fever, pulse 85, tongue clean.

*July 1st.*—Slight swelling, pulse 95, tongue furred, wound discharges small quantity of reddish serum, and has an erysipelatous look. ℞. Lotio nitrat plumbi. Elevate forearm, plaster and roller removed.

*July 2d.*—Great tension of the whole arm, high fever; removed stitches from the wound, and applied a yeast and Peruvian bark poultice. ℞. Pil. opii gr. ii.

*July 3d.*—Better; fever abating; odor less offensive.

*July 4th.*—Erysipelatous appearance has declined in a measure; fever abating; has had retention of urine from the effects of opium, which the house student attempted to relieve by trying to pass a catheter, in which he failed; patient was entirely relieved by warm fomentations, and a large, warm stimulating enema.

*July 6th.*—Wound granulating; poultice discontinued, and a weak solution of nitrate of lead applied; from this date the patient gradually improved; the wound healed without any soreness, and to his great gratification he gradually regained the use of the flexor and extensor muscles, whose functions had been almost entirely impaired, to that extent that he could not close his hand, and had but slight motion of the wrist or fingers. When last heard of he was using his arm as freely as the sound one.

## Figures and Tables

**Figure f1:**
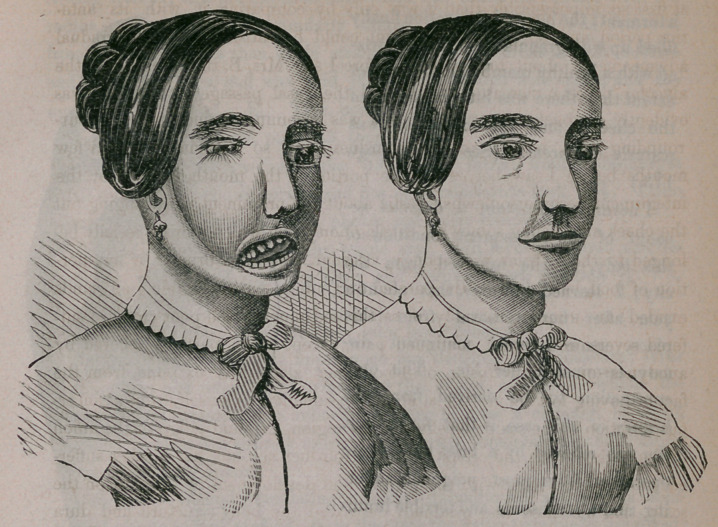


**Figure f2:**